# The Optimization and Analysis of a Triple-Fin Heterostructure-on-Insulator Fin Field-Effect Transistor with a Stacked High-k Configuration and 10 nm Channel Length

**DOI:** 10.3390/nano13233008

**Published:** 2023-11-23

**Authors:** Priyanka Saha, Rudra Sankar Dhar, Swagat Nanda, Kuleen Kumar, Moath Alathbah

**Affiliations:** 1 Department of ECE, NIT Mizoram, Aizawl 796012, Indiananda.swagat@gmail.com (S.N.); kuleen.elx@gmail.com (K.K.); 2 Department of Electrical Engineering, College of Engineering, King Saud University, Riyadh 11451, Saudi Arabia

**Keywords:** TG FinFET, physical oxide thickness HOI device, stacked high-k, Silvaco TCAD, V_TH_, DIBL

## Abstract

The recent developments in the replacement of bulk MOSFETs with high-performance semiconductor devices create new opportunities in attaining the best device configuration with drive current, leakage current, subthreshold swing, Drain-Induced Barrier Lowering (DIBL), and other short-channel effect (SCE) parameters. Now, multigate FETs (FinFET and tri-gate (TG)) are advanced methodologies to continue the scaling of devices. Also, strain technology is used to gain a higher current drive, which raises the device performance, and high-k dielectric material is used to minimize the subthreshold current. In this work, we used stacked high-k dielectric materials in a TG n-FinFET with three fins and a 10 nm channel length, incorporating a three-layered strained silicon channel to determine the short-channel effects. Here, we replaced the gate oxide (SiO_2_) with a stacked gate oxide of 0.5 nm of SiO_2_ with a 0.5 nm effective oxide thickness of different high-k dielectric materials like Si_3_N_4_, Al_2_O_3_, ZrO_2,_ and HfO_2_. It was found that the use of strained silicon and replacing only the SiO_2_ device with the stacked SiO_2_ and HfO_2_ device was more beneficial to obtain an optimized device with the least leakage and improved drive currents.

## 1. Introduction

A FinFET device is a perpendicular-oriented device with multiple regions and is surrounded by conductor gate material. The gate creates electrostatic control over nearly the entire portion surrounding the device, providing efficient control over the inverted nano-scaled channel [1]. In terms of performance, this results in an abrupt subthreshold slope upon scaling, meaning higher figure sensitivity. Due to its ability to perform exact assessments of technical progress, a FinFET also exhibits high consistency and repeatability [2]. The utilization of a FinFET is the standard solution to eliminate SCEs. At the nanoscale level, Heterostructure-on-Insulator (HOI) FinFETs [3,4,5] are preferred to enhance drain current. In HOI FinFETs, the fin is created on buried oxide (BOX), and it boosts the elimination of dependent capacitances and paired difficulty [6]. To continue Moore’s law, FinFETs are a better option. The 3D design of FinFETs permits them to be used as multigate devices. It is relatively possible to produce FinFETs using bulk and HOI technology where the gate length used is 10 nm in the fabrication design. In a bulk FinFET, the fin area is formed using the fin definition. In FinFETs, to increase the off current and boost the current drive per fin, the oxide thickness should be narrowed, and several performance aspects are focused on: (i) alternation of the effective oxide thickness, (ii) the use of dielectric material like high-k materials, (iii) the elevation of channel doping, and (iv) the incorporation of the strained silicon methodology in a device. Furthermore, a different approach was introduced in device design by increasing the fin count [7] in the particular structure and optimizing the fin heights and fin proportions to achieve a trade-off in device active currents. Hence, different architecture-based FinFETs have been developed, such as single-fin FinFETs, double-fin FinFETs, triple-fin FinFETs, and quad-fin FinFETs. FinFETs are replacing regular planar structures or double-gate field-effect transistors due to their scaling down in channel length, better electrostatic control over approximately one-third of the channel region, and occupation of less die area [8], leading to low power dissipation.

Since the beginning of CMOS technology, SiO_2_ has been the only material for gate dielectrics. Scaling SiO_2_ exposes its elementary restrictions related to exponential increases in gate leakage, process controllability, and consistency [9]. When the thickness of the gate oxide drops, and an abrupt increase in the subthreshold current occurs due to SiO_2_ channeling; an alternate material for the oxide layer region (i.e., SiO_2_) has to be introduced along with the high-k materials to diminish the leakage current of the gate in Si/SiGe/Si channel HOI devices. In the strain methodology, a modification is made to the MOSFET by growing a heterostructure channel with Si/SiGe/Si implanted within the structure. HOI-strained silicon works enormously well, along with there being an improvement in the drive current.

Recent demonstrations of HOI-strained silicon channel FinFETs with stacked high-k suggest that flexible strain in the strained Si layer increases the current and facilitates electron transportation along the channel’s orientation. However, as FinFETs are scaled down, a major hindrance is observed in the form of surface roughness scattering. The roughness at the interfaces affects electron conduction in the channel, influencing electron mobility [10] and degrading drain currents. For generations, an equivalent oxide thickness (EOT) has been applied with various high-k gate dielectric materials [11,12]. On the other hand, when high-k dielectric materials are directly placed on silicon, the device performance could decline. Due to reduced interface eminence and development compatibility as well as emerging difficulties, such as stability and reliability, the device presentation needs to be upgraded. A high-k dielectric is used in place of the gate oxide, whereas an alternative gate stack (GS) method with a thin interfacial SiO_2_ layer is the obvious solution to resolve this problem [13,14,15,16,17,18]. To moderate this problem, a gate stack (GS) configuration has been introduced. In the GS structure, a very thin layer of SiO_2_ is initially positioned on silicon to strengthen the interface quality, and then, high-k dielectrics are placed over the SiO_2_ layer to decrease the gate tunneling current.

Currently, arrays of fins in FinFETs [19] are being realized in today’s technical era for higher switching speeds in digital circuits. Though, with silicon or with Silicon-on-Insulator (SOI) array FinFETs or HOI array FinFETs [20] that are incorporated in various devices, there is a determination to meet the requirements set by the International Roadmap of Devices and Systems (IRDS) 2022 for the 3 nm technology node [21]. Multi-fin FETs have a higher packaging density compared to Gate-All-Around Field-Effect Transistors (GAAFETs) because there are still challenges surrounding pitch scaling in design technology and area reduction, where process-related dimension control is required. The 3D stack structure, as a future IRDS proposal, is therefore not yet considered to have enhanced performance in the nano regime. Researchers are characterizing and analyzing device scaling to meet these challenges, particularly the fabrication process of GAAFETs on a single silicon substrate. Therefore, to build a faster and optimized device, the existing and developed FinFET technology is implemented here since there is still a requirement to reach the proposed performance of a 3 nm technology node, as per IRDS 2022. Hence, the need arises to design and optimize a high-k stacked three-fin FinFET nanodevice system to meet the requirements of optimized and enriched performance.

Bha et al. [22] designed FinFET devices with a channel length of 10 nm on the buried oxide layer, which showed reduced leakage current and high transconductance. Thereafter, Nanda et al. [23,24] simulated a channel-engineered TG FinFET with a channel length of 10 nm and found the device characteristics to be on par with the 3 nm technology node with a strained-silicon channel system. The device showed an efficient reduction in the SCEs and better device performance. Even though the on current is improved when using a strained-silicon channel, achieving the requirement of high speed with minimal power consumption, there is a need to increase the total performance further by implementing stacked high-k dielectrics in a HOI structure; hence, developing a three-fin stacked high-k HOI FinFET device system is the consensus.

In the present work, a TCAD mixed-mode simulator is used to compare and analyze the SCEs incorporating a high-k dielectric material in a three-fin TG FinFET device. The GS configuration is used throughout the study. This paper presents an analysis of three-fin gate stack high-k dielectric material-based strained-channel HOI FinFETs along with quantum analysis results from the optimized structure detected here.

## 2. Device Structure

The characteristics of a MOSFET are well explained by channel engineering, which is applied in various MOSFETs, where the channel electric field, *E_x_*, is reduced appreciably compared with the vertical electric field implementing Poisson’s equation in 1D form. So, *W_C_* throughout the channel is specified by the potential in 1D, but this estimation is best suited when the gate length, *L*, is bigger than *W_C_*.

Following the estimation made, the inversion layer charge is observed to be small enough in the channel region and, hence, the charge is given as:

(1) $$Q_{in}=C_{ox}(V_{gs}-V_{TH}-V_{pc,s})$$

where $V_{pc,s}$ is the channel potential with respect to the source. In the source, $V_{pc,s}=0$, and in the drain, $V_{pc,s}=V_{ds}$. In the source end and drain end, X = 0 and X = L are added simultaneously.

The drain current in the channel, *I_Dr_*, is the drift current instigated by the electric field *E_x_* in the channel:

(2) $$I_{Dr}=\frac{Q_{in}W_{C}L_{C}}{t_{C}}$$

where *W_C_* is the width of the channel and *t_C_* is the time of carrier transit.

*Q_in_* does not decrease to zero when *V_gs_* ≤ *V_TH_*. In the off-state condition, the leakage current generated is unwanted because it degrades the device performance based on the current flowing through the device. This leads to conversion from the depleted channel to the inverted channel for uniform *V_gs_*, which is the subthreshold voltage. The drain current acquired at this point, which holds on for *V_gs_* ≤ *V_TH_*, is the subthreshold current. The subthreshold drain current is, therefore, specified by:

(3) $$I_{Dr}=\mu_{eff}C_{ox}\frac{W_{C}}{L_{C}}\left(n-1\right){\left(\frac{KT}{e}\right)}^{2}e^{\frac{q\left(V_{gs}-V_{TH}\right)}{nKT}}\left(1-e^{-eV_{ds}/KT}\right)$$

When the drain voltage is greater than *KT*/*e*, $(1-e^{-eV_{ds}/KT})\approx 1$, so the following is acquired:

(4) $$I_{Dr}=\mu_{eff}C_{ox}\frac{W_{C}}{L_{C}}\left(n-1\right){\left(\frac{KT}{e}\right)}^{2}e^{\frac{q\left(V_{gs}-V_{TH}\right)}{nKT}}$$

It is obvious that the subthreshold current is free from *V_gs_* − *V_ds_* and decays gradually with decreasing *V_gs_*. The *I_off_* is usually described as the I_D_ current when *V_gs_* = 0 and *V_ds_* = *V_dd_*. It is defined as *I_Dr_*. This *I_off_* is calculated considering certain parameters such as the physical dimension of the channel, depth of the source or drain junction, gate oxide width, doping concentration for the channel or surface, and *V_dd_*. *I_off_* is expected to increase by nearly 10 times for a 100 mV decay of *V_TH_*.

The charge carrier mobility, *μ_eff,n_* and *μ_eff,p_*, in the inversion region can be defined via the following general equations [25]:

(5) $$\mathrm{For}\;\mathrm{the}\;\mathrm{electron}\;\mathrm{mobility},\;\mu_{eff,n}=\frac{638}{1+{(E_{eff}/{7\times 10}^{5})}^{1.69}}$$

(6) $$\mathrm{For}\;\mathrm{the}\;\mathrm{mobility}\;\mathrm{for}\;\mathrm{hole},\;\mu_{eff,p}=\frac{240}{1+(E_{eff}/{2.7\times 10}^{5})}$$

where *E_eff_* is the effective channel electric field of the device. This can be observed as *μ_eff_* declining very fast with increasing *E_eff_*.

In cases where *V_gs_* > *V_T_*, *I_Dr_* is given by:

(7) $$I_{Dr}=\frac{W_{C}}{L_{C}}\mu_{eff}C_{ox}\left[{(V}_{gs}-V_{TH}\right)V_{ds}-\frac{n{V_{ds}}^{2}}{2}$$

In a linear system, the *I_D_* can be stated as:

(8) $$I_{Dr}=\frac{W_{C}}{L_{C}}\mu_{eff}C_{ox}\left[{(V}_{gs}-V_{TH}\right)V_{ds}$$

When *V_ds_* is increased further, the increase in *I_Dr_* seems to be parabolic in nature and is given by:

(9) $$I_{Dr}=I_{D,sat}=\frac{W_{C}}{L_{C}}\mu_{eff}C_{ox}\frac{{\left(V_{gs}-V_{TH}\right)}^{2}}{2n}$$

(10) $$I_{Dr}=\mu_{eff}C_{ox}\frac{W_{C}}{L_{C}}\left(n-1\right){\left(\frac{KT}{e}\right)}^{2}e^{\frac{e\left(V_{gs}-V_{TH}\right)}{nKT}(1-e^{-eV_{ds}/KT})}$$

where $n=1+C_{dep}/C_{ox}$.

A linear plot of ln(*I_D_*) as a function *V_gs_* in the subthreshold region is achieved and the slope for subthreshold swing (SS) is attained as a degree of the efficiency of *V_gs_* in *I_Dr_* modulation. A minor slope for subthreshold is, hence, needed for converting the off current for the transistor. In low *V_gs_*, the current decreases from the subthreshold region to the off-current state. This controls the *I_off_* current and the power dissipation in the device circuitry. 

For *V_gs_* < *V_TH_*, one can describe the subthreshold region using a direct equation of drain current in the subthreshold regime for a double gate, given by:

(11) $$I_{D}=\frac{W_{C}}{L_{C}}\mu_{n}4C_{si}{\left(\frac{KT}{q}\right)}^{2}\left(1-e^{\left(-\frac{q}{KT}\right)\left(\frac{Q_{D}}{8C_{si}}\right)}\right)\cdot \left[1-e^{-\left(\frac{qV_{ds}}{KT}\right)}\right]\cdot e^{q/KT(V_{gs}-V_{FB}-\left[\frac{\frac{Q_{D}}{2}}{C_{ox}}\right]-2\varphi_{f})}$$

where *μ_n_* = electron mobility as *C_Si_* = *ε_Si_*/*t_Si_* is the thin film on silicon, while *V_ds_* and *V_gs_* are the drain-to-source and gate-to-source voltages, respectively. The difference in *I_Dr_* current of the device with respect to the difference in *V_gs_* at the subthreshold region gives the subthreshold slope as:

(12) $$SS=\frac{\delta V_{gs}}{\delta (logI_{d})}=\frac{\delta V_{gs}}{\delta \varphi_{S}}\cdot \frac{\delta \varphi_{S}}{\delta (logI_{d})}$$

where $\frac{\delta V_{gs}}{\delta \varphi_{S}}$ = the m-factor for the body and defines the coupling between the gate and the surface potential, though $\frac{\delta \varphi_{S}}{\delta (logI_{D})}$, called the n-factor, is incomplete for a minimum value that corresponds to the Fermi–Dirac distribution. For a bulk MOSFET, the subthreshold slope can further be expressed as:

(13) $$SS=n\cdot m=\frac{KT}{q}\ln\left(10\right)\left(1+\frac{C_{D}+C_{it}}{C_{ox}}\right)=59\left(1+\frac{C_{D}+C_{it}}{C_{ox}}\right)mV/dec$$

where *C_D_* and *C_it_* = capacitances in the depletion region and the trap interface states, respectively. The SS is constant and also not dependent on either *V_gs_* or *V_ds_*. An ideal FET has an *SS*_0_ = 59 mV/dec at room temperature (300 K). Approaching the ideal value, the full-depletion set-up agrees with the thin-film system developed here. The charge varies with depletion at the front gate and is given as *δQ_Dr_*/*δV_gs_* = 0, meaning that *C_D_* ≈ 0 and the *SS*, therefore, obtains its theoretical value with *m* = 1, so the subthreshold slope is given as

(14) $$SS≅\frac{KT}{q}\log\left(10\right)\equiv {SS}_{0}$$

In downscaling, for a thin-film device with the same parameters as a bulk device or thick-film device, the subthreshold slope will be steeper. Every change in the gate voltage, *V_gs_*, in the subthreshold domain is, therefore, precisely linked to the surface potential, *φS*, resulting in an identical rise in both the variables due to the traps at the Si-oxide interface contact, and the theoretical limit is never met in a practical device.

A number of three-fin tri-gate FinFETs were developed here involving different channel oxides by replacing the regular SiO_2_ layer with different high-k dielectric materials such as Si_3_N_4_, Al_2_O_3_, ZrO_2_, and HfO_2_, which were then stacked on the existing SiO_2_ separately considering the different equivalent oxide thickness (EOT) for the same physical thickness and low leakage for a dielectric. The equivalent oxide thickness calculations (15) are shown as follows [26]:

(15) $$EOT=t_{high\text{-}k}\frac{k_{{SiO}_{2}}}{k_{high\text{-}k}}$$

where $t_{high\text{-}k}$ = the high-k material’s physical thickness, $k_{{SiO}_{2}}$ = the SiO_2_ dielectric constant, and $k_{high\text{-}k}$ = the high-k material dielectric constant. 

**Figure 1:**
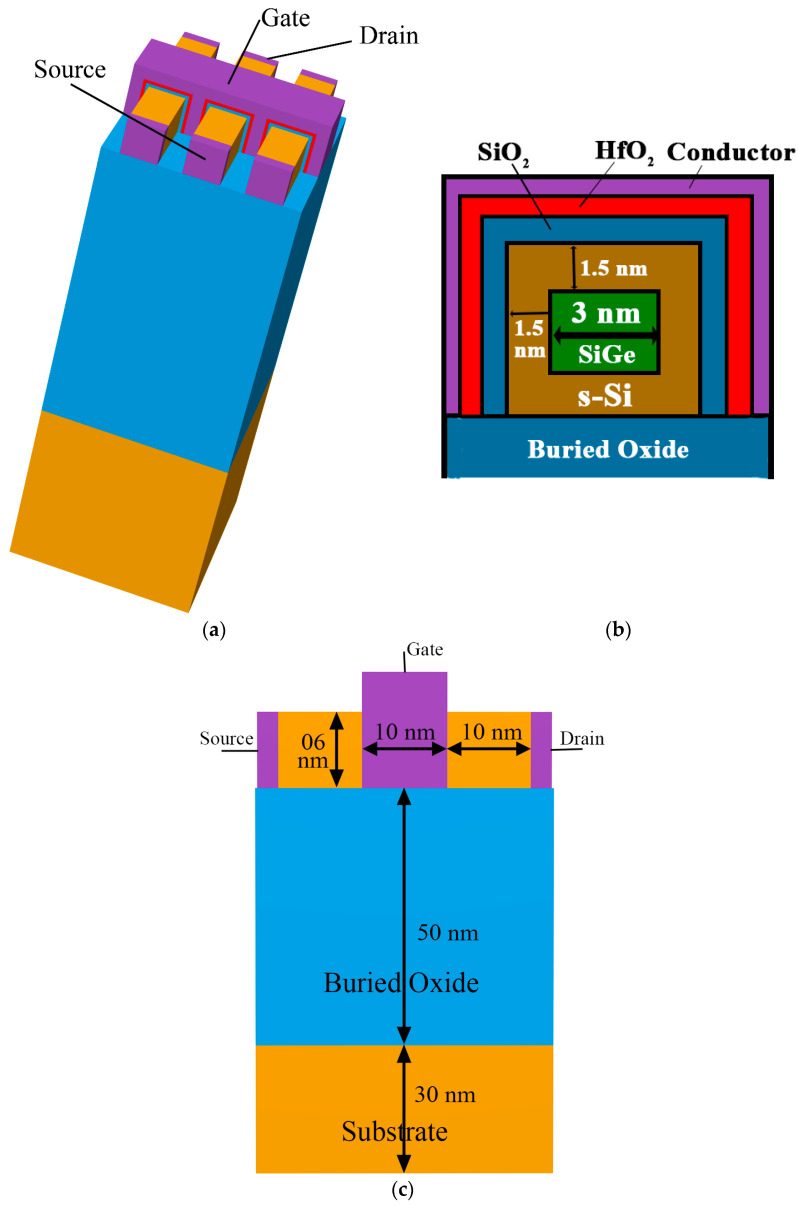
(**a**) Three-fin FinFET structure, (**b**) inset of the cross-section of a single fin, and (**c**) dimensional representation of the three-fin HOI FinFET.

TG FinFETs containing different high-k dielectric materials are expected to be immensely beneficial for providing low off currents for the proposed device and, hence, an improvement in the device characteristics is expected. The device schematic was adjusted for a 10 nm channel length and is presented in Figure 1. The physical width of the channel oxide was kept constant. Keeping the 1 nm physical thickness of the stacked high-k dielectric material fixed, the device modification was carried out for different high-k amounts in the device. Here, in this structure, to maintain the same physical thickness, 0.5 nm of high-k dielectric material over the channel region of the device was added on top of 0.5 nm of SiO_2_, which was used as the stack gate oxide. The physical thickness of the high-k dielectric material layer used was 0.5 nm; hence, the total physical thickness was 1 nm, while the EOT was analyzed to determine the effective gate oxide thickness in the device. When using strained technology along with a high-k stack and a tri-layered silicon channel nanosystem in a three-fin FinFET, the control of the short-channel parameters is expected to be highly beneficial. The SiGe layer is placed in between two silicon layers, and a strained silicon region is developed, primarily forming a three-layered channel. As a result, the device is expected to provide enhanced performance due to induced strain in the channel for the 10 nm channel length FinFET, while improved off current is expected by controlling the SCEs through high-k gate oxide stack systems, which is the motivation of this paper.

The dimension specifics of the device are summarized in Table 1, where the structure is presented placing three different layers on the channel. In the 10 nm three-fin HOI TG FinFET, the 1.5 nm thick silicon layer of the channel is strained and is displayed along with the 3 nm thick SiGe layer. Analysis of the device performance was carried out using Silvaco Atlas TCAD tools [27]. The structure of the stacked high-k three-fin FinFET with a tri-layered strained silicon channel is displayed in Figure 1. The physical dimensions and the EOT of all the high-k dielectrics used in the paper are tabulated in Table 2.

## 3. Results and Discussion

**Figure 2:**
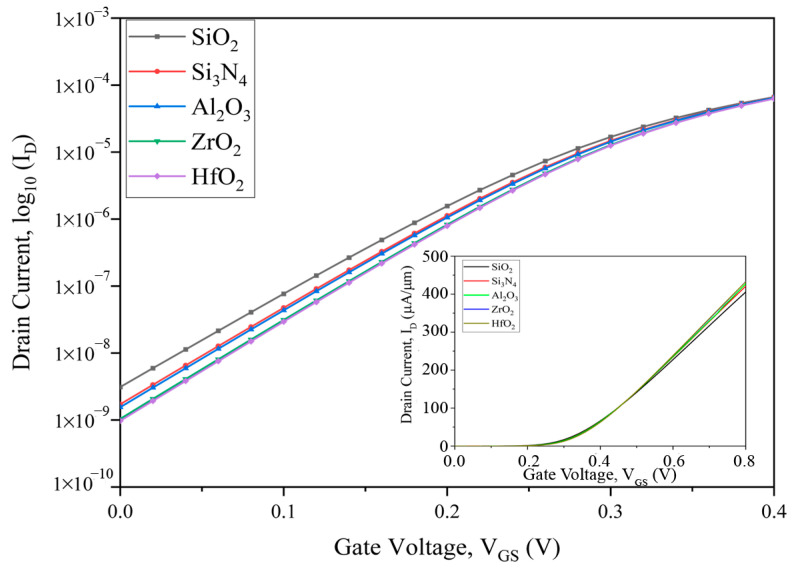
*I_D_*–*V_gs_* transfer plot of three-fin TG FinFETs on a log scale at *V_ds_* = 1.0 V.

The linear (inside) and logarithmic characteristics graph of the stacked high-k three-fin strained TG FinFET is shown in Figure 2. The drain current versus gate voltage transfer plots of the stacked high-k three-fin strained FinFETs with different stacked high-k gates are compared and plotted in Figure 2, where the HfO_2_-based devices show better performance. The logarithmic plot of drain current versus gate voltage displays the off current and SCE parameters for all the devices.

**Figure 3:**
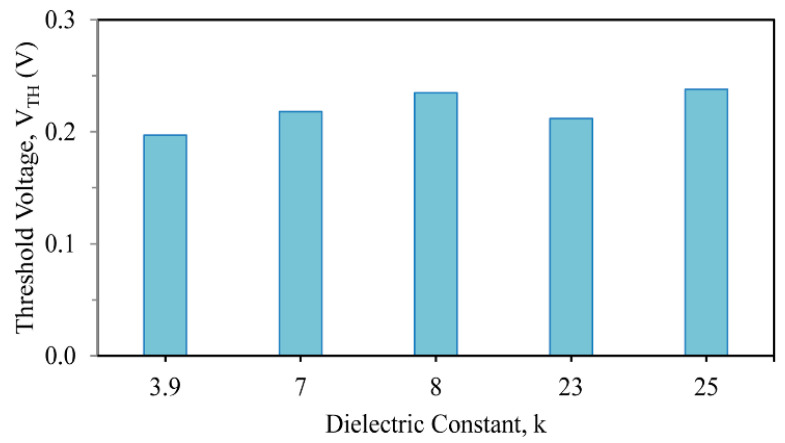
Different threshold voltages (V_TH_) of stacked 3-fin TG strained FinFETs with several gate dielectrics.

The threshold voltages (V_TH_) of the developed devices with SiO_2_ added to the high-k material (like Si_3_N_4_, Al_2_O_3_, ZrO_2_, and HfO_2_) to form the stack gate for the 10 nm three-fin TG FinFETs are calculated to be 0.218, 0.235, 0.212, and 0.238 V, respectively, as shown in Figure 3. The device with only a SiO_2_ gate has a threshold voltage of 0.197 V. A comparison of only the SiO_2_ dielectric and stacked high-k dielectric materials was made, and it was observed that the HfO_2_-based device had the highest threshold voltage; hence, a replacement for SiO_2_ and other stacked high-k materials should be considered in the stacked arrangement for three-fin FinFET devices to provide improved voltage control.

**Figure 4:**
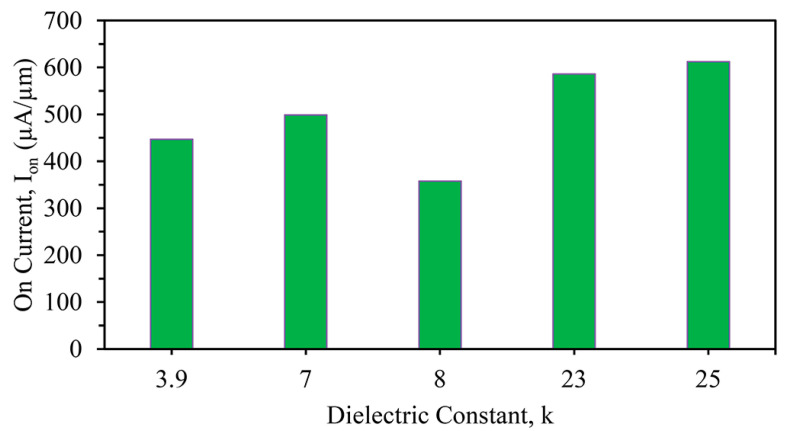
Different on currents (*I_on_*) of stacked three-fin TG strained FinFETs with several gate dielectrics.

The on current (*I_on_*) was 446.54 μA/μm for the SiO_2_-only device, whereas 498.90, 357.73, 585.92, and 612.24 μA/μm were observed, respectively, for the Si_3_N_4_-, Al_2_O_3_-, ZrO_2_-, and HfO_2_-based devices. The maximum on current was exhibited for the HfO_2_ stacked high-k device due to the high dielectric value with an average work function, as seen in Figure 4. This difference was due to the use of several gate metals in the three-fin device, in which the work functions were variable. The maximum on current (I_on_) is clearly shown in Figure 4.

**Figure 5:**
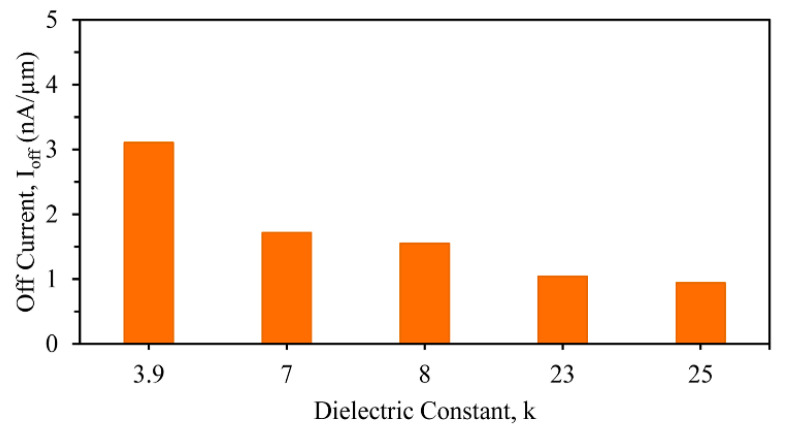
Different off currents (*I_off_*) of stacked three-fin TG strained FinFETs with several gate dielectrics.

Thereafter, an analysis of the characteristics of the different material-based gate dielectric devices, like subthreshold swing, off current (*I_off_*), and DIBL, was carried out to conclude which dielectric is suitable for the three-fin optimized device. The off current can be calculated using the below-mentioned formula [28]:

(16) $$I_{off}(nA)=100\frac{w}{L}10^{-V_{TH}/SS}$$

where *W* = the width of the channel and *L* = the length of the channel, *V_TH_* = the threshold voltage, and *SS* = the subthreshold swing. The off current (*I_off_*) variations for different gate dielectrics with high-k stacks were 0.94, 1.72, 3.11, 1.04, and 1.55 pA/μm for HfO_2_, Si_3_N_4_, SiO_2_, ZrO_2,_ and Al_2_O_3_, respectively, as displayed in Figure 5. For improved device characteristics, the off current should be as low as possible, and it was found that the device with a SiO_2_ and HfO_2_ gate stacked oxide combination was the best alternative.

**Figure 6:**
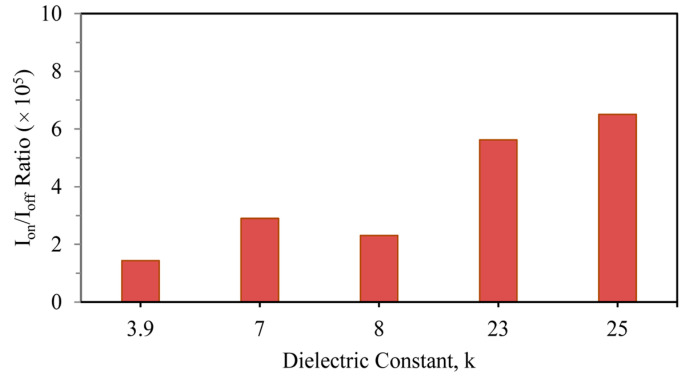
Different *I_on_*/*I_off_* ratios of stacked three-fin TG strained FinFET with several gate dielectrics.

Next is the comparison of the *I_on_*/*I_off_* ratio, which is shown in Figure 6. For enhanced device performance, this ratio needs to be as high as possible. The *I_on_*/*I_off_* factors for SiO_2_, Si_3_N_4_, Al_2_O_3_, ZrO_2_, and HfO_2_ were observed to be 1.44, 2.90, 2.31, 5.62, and 6.51 (×10^5^), respectively. So, the device structure with HfO_2_ fulfils the requirement of providing the maximum on current with minimum leakage, so it can be concluded that the HfO_2_ dielectric material as the gate oxide is the most suitable in a stacked three-fin strained-channel FinFET device.

**Figure 7:**
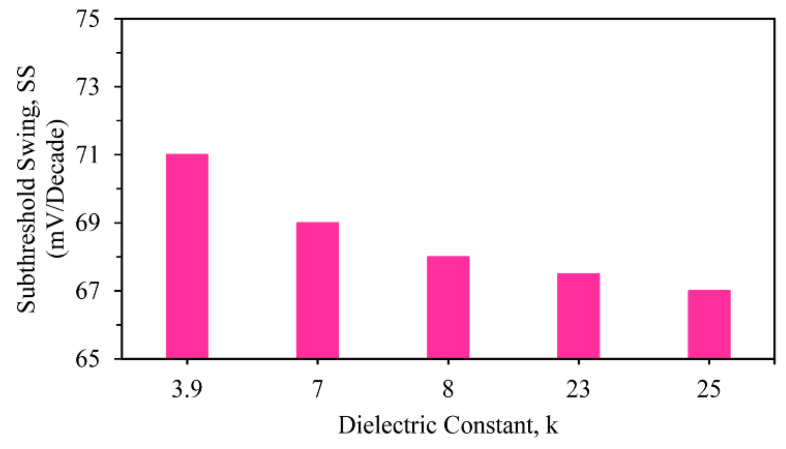
Different SSs of stacked three-fin TG strained FinFET with several gate dielectrics.

The next parameter, subthreshold swing, was analyzed using a linear plot, as shown in Figure 7. Here, the value of subthreshold swing of the SiO_2_ with HfO_2_ was 67 mV/decade, whereas it was 71.05 mV/decade for the SiO_2_-only device, thereby clearly showing that the stacked three-fin TG strained FinFET of SiO_2_ + HfO_2_ achieved enhanced performance in comparison to the others. For *SS* calculation, we applied the following equation [28]:

(17) $$SS\left(\frac{mV}{dec}\right)=\frac{dV_{gs}}{d({log}_{10}\left(I_{DS}\right))}$$

where *dV_gs_* = the shift in the gate voltage and *d*(*log*_10_(*I_DS_*)) = the shift in the logarithmic drain current. The variations in the *SS* of different high-k stacked three-fin TG strained FinFETs are displayed in Figure 7.

**Figure 8:**
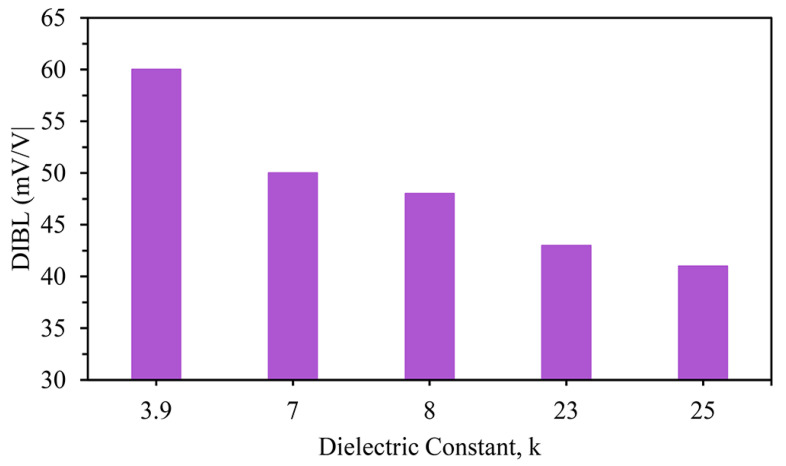
Different DIBL values of stacked three-fin TG strained FinFETs with several gate dielectrics.

The last-compared factor presented here is the DIBL, and the results are shown in Figure 8: 59.73, 49.78, 48.65, 42.90, and 40.99 mV/V for the SiO_2_-only gate oxide material followed by the high-k stacks of Si_3_N_4_, Al_2_O_3_, ZrO_2_, and HfO_2_, respectively. The lowest DIBL value, and hence the best performance, was observed for the HfO_2_-based device, as displayed in Figure 8.

As a result, it is evident that the high-k dielectric material with a stacked arrangement of 0.5 nm of SiO_2_ and HfO_2_ of 0.5 nm physical thickness is the best fit to substitute SiO_2_ for managing SCEs at a channel length of 10 nm.

This demonstrates the effects of creating an enhanced three-fin technology device incorporating HfO_2_-based high-k material to improve the performance and meet the IRDS 2022 specifications for 3 nm technology node data.

Table 3 shows a detailed comparison, indicating better threshold voltage, on current, off current, SS, and DIBL for the 10 nm HOI high-k (HfO_2_) stack than the existing 10 nm HOI three-fin FinFET; therefore, the former is considered to have enhanced performance in comparison to the standards suggested by IRDS 2022 [21].

The 10 nm HOI high-k (HfO_2_) stack is reported to be adequate, despite the 82 mV/decade subthreshold swing (SS) as per IRDS 2022 for HP devices.

### Quantum Results

**Figure 9:**
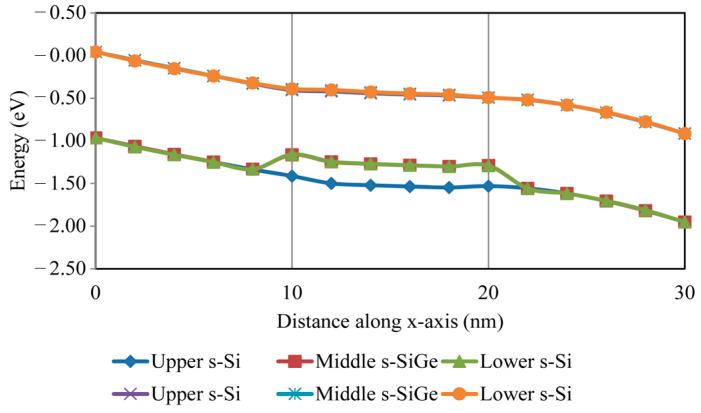
Energy band formation in FinFET structure.

In the gate, electrons are exhibited with regards to the semiconductor, and holes in the semiconductor are exhibited with regards to the gate. Figure 9 displays an energy band diagram of the accumulation region. In the semiconductor region, the band-bending curves were observed to rise. The middle s-SiGe and lower s-Si near the HOI structure are much closer to the conduction band than the valence band. From Figure 9, it can be seen that the band cutline occurs in the strained-channel regions, where charge carriers are narrowed and confined towards the s-SiGe level, owing to the development of a quantum well arrangement in the channel. In the tri-layered channel, the effective mass due to the bandgap is reduced with the increased mobility of the carriers along with the biaxial strain incorporated into the channel region, which in turn affects the band bending in the quantum barrier of the channel in the nano-regime.

**Figure 10:**
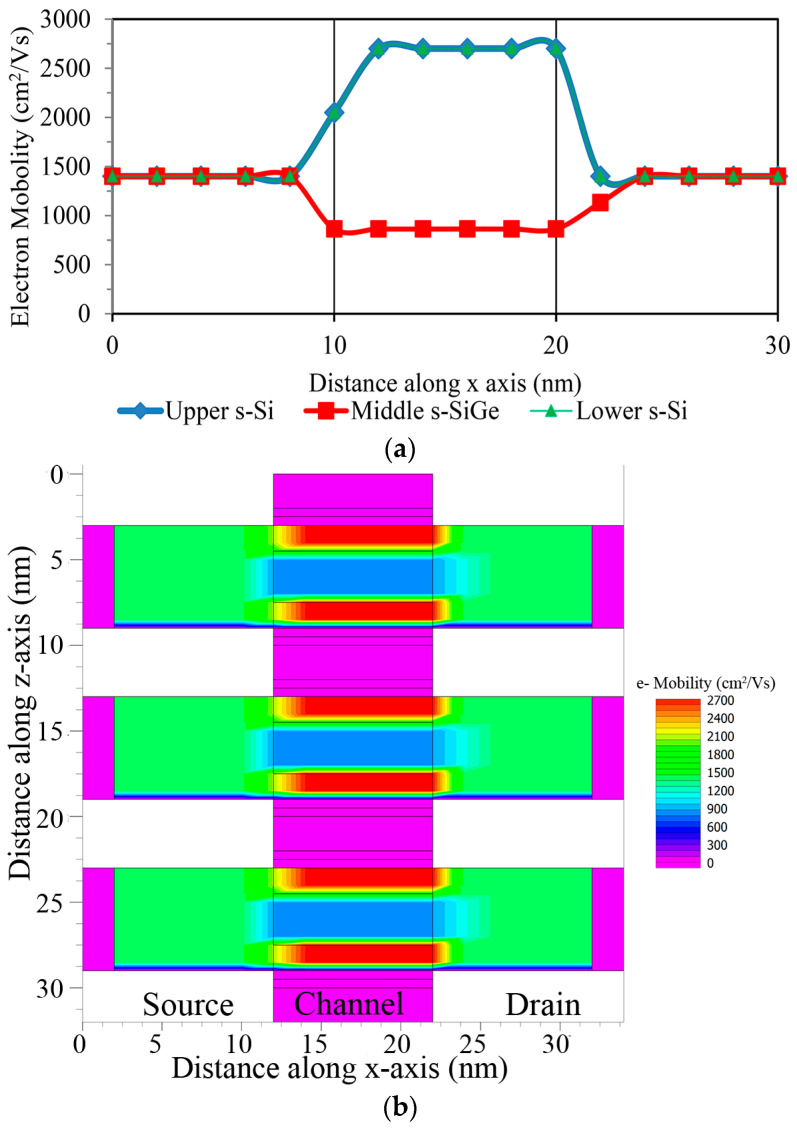
(**a**) Electron mobility variation in tri-layered channel; (**b**) electron mobility contour along the lateral length.

Owing to the reduced channel dimensions due to the effective mass, mobility is improved for the tri-layered strained silicon channel device. The currently developed device with an interfacial layer thickness of less than 2 nm and hafnium-based dielectrics has inferior electron mobility than the device with only a SiO_2_ dielectric. The mobility exhibits more degradation via remote phonon scattering in the HfO_2_ dielectric than the SiO_2_ one, which can be successfully isolated by presenting a stacked high-k system in the tri-layered n-FinFET strained silicon technology device. Due to their quantization, the effective masses and charge carriers degenerate the strained silicon in the channel region, and, therefore, the mobility begins to increase. In the case of a tri-layered strained technology structure where high-k materials are used as the gate dielectric, the mobility is reduced to ~850 cm^2^/Vs in the middle s-SiGe layer, whereas it increases to 2700 cm^2^/Vs in the lower and upper s-Si layers, as clearly observed in Figure 10a; Figure 10b shows the mobility variation contour diagram. It was found that the HfO_2_ dielectric was significantly more affected by remote phonon scattering than the SiO_2_ dielectric, since the dielectric constant was high. From the electron mobility contour diagrams, increased electron mobility can be seen in the lower s-Si across the QW channel length, which is a strain-induced nano-regime structure with a 10 nm gate length for the 3 nm technology node, initiating quantum tunneling via ballistic transport using the s-SiGe well region. As a result of the shorter channel length (10 nm) of the device, ballistic transport occurs and only minor scattering roughness is observed in the system, which is undoubtedly witnessed in the mobility contours (Figure 10b), indicating a smooth passage for electrons in the device across the strained-Si layer.

**Figure 11:**
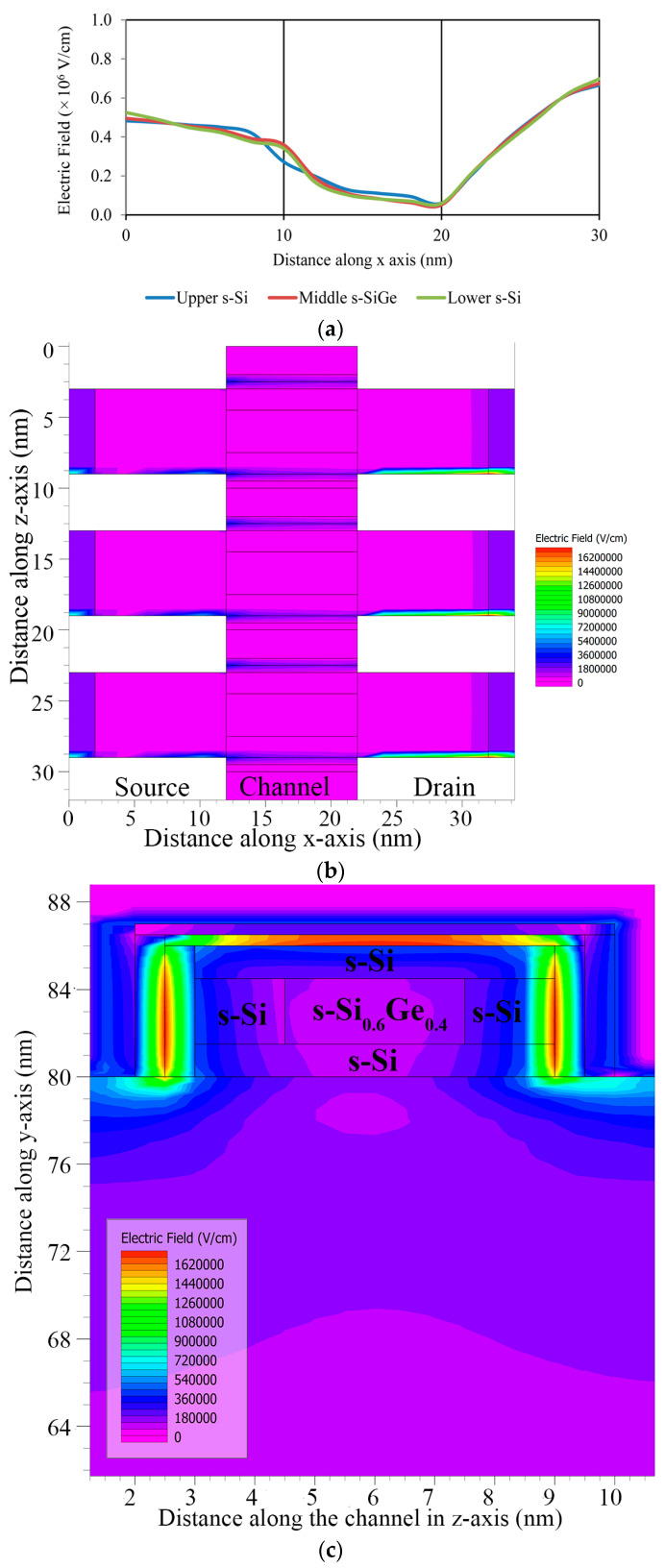
(**a**) Electric field analysis along source to drain. (**b**) Electric field contour along lateral length. (**c**) Electric field contour along fins’ width.

From the drain to source region, the electric field passes, allowing quantum carriers with minor constraint in the s-SiGe well region of the hetero-band structure channel in the quantum well barrier system to create a tunneling path, as shown in Figure 11a. The contour for the electric field in the 10 nm gate length FinFET device is displayed in Figure 11b,c. An extremely high electric field is noticeable due to the reduced gate length structure with an informal doped channel that introduces velocity saturation in the device. These interpretations are attributed to the occurrence of quasi-ballistic carrier transport because of the gate length limitation, which is induced by carrier regulation in the strain-induced thin s-Si well region of the quantum well barrier nanosystem device. This ballistic transport condition with carrier confinement is recognized in the HOI structure combination of the nano-channel device, which leads to the tunneling of the quantum charge carriers. 

**Figure 12:**
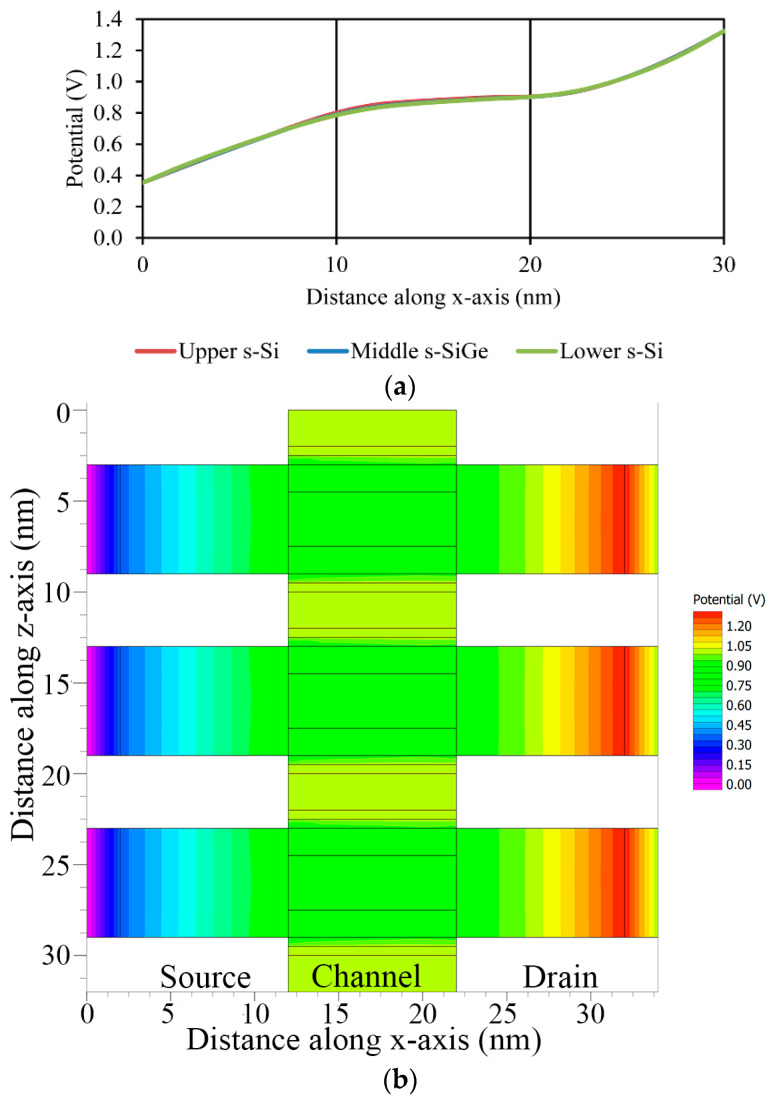

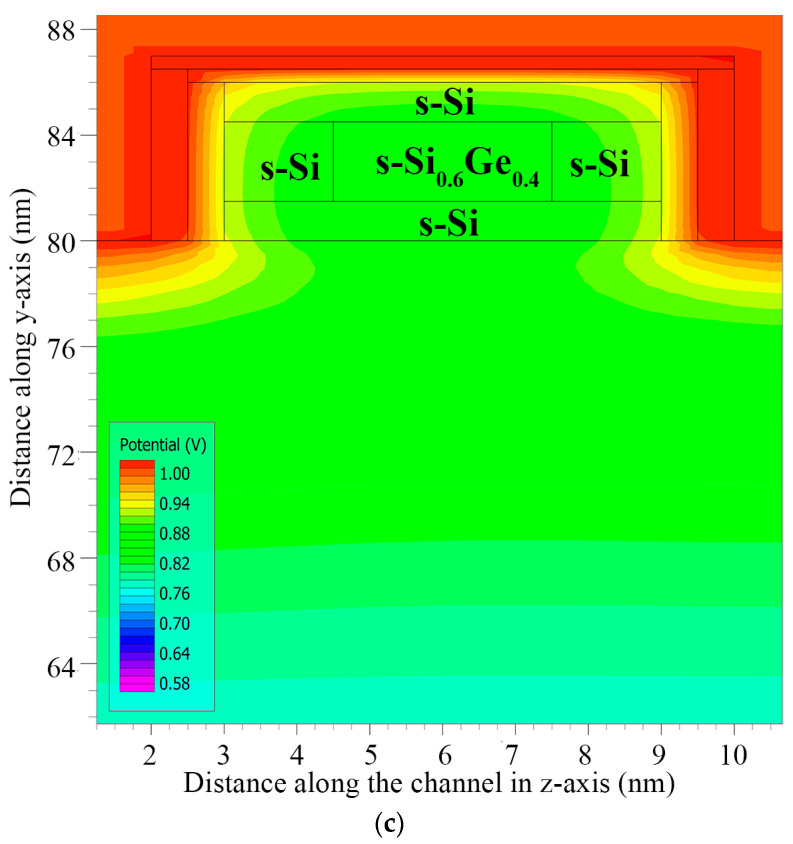
(**a**) Electric potential along the channel length. (**b**) Potential contours along length. (**c**) Potential contours along width.

Concentrating on bandgap moderation, extreme electrostatic potential changes through s-SiGe deposition, concerning *V_gs_* for 1 V, were attained. The tri-gate FinFET with stacked high-k material established had a 10 nm channel length with 10^18^ cm^−3^ doping for the source and drain region; however, the tri-layered strained HOI quantum well barrier channel was moderately doped with a concentration of 10^15^ cm^−3^. The potential observed close to the source and the drain end increased with the increase in the drain voltage (*V_ds_*), causing the potential of the inside strained layers to develop in the channel of the structure. The potential graph is shown in Figure 12a, and a detailed analysis of the potential contours is displayed in Figure 12b,c.

**Figure 13:**
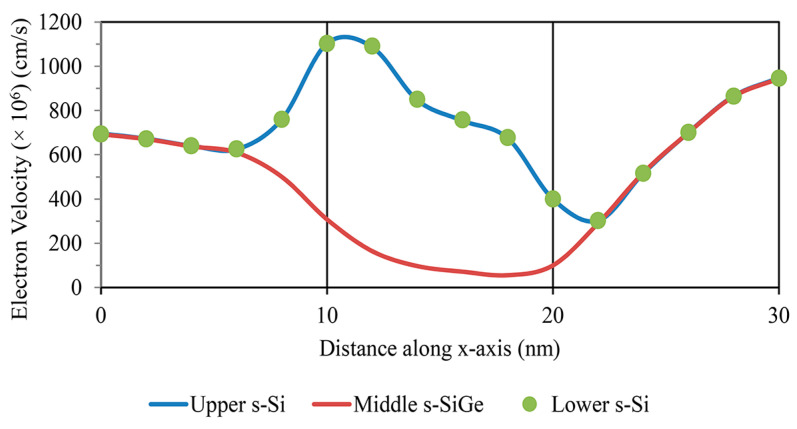
Electron velocity variation along the channel length.

**Figure 14:**
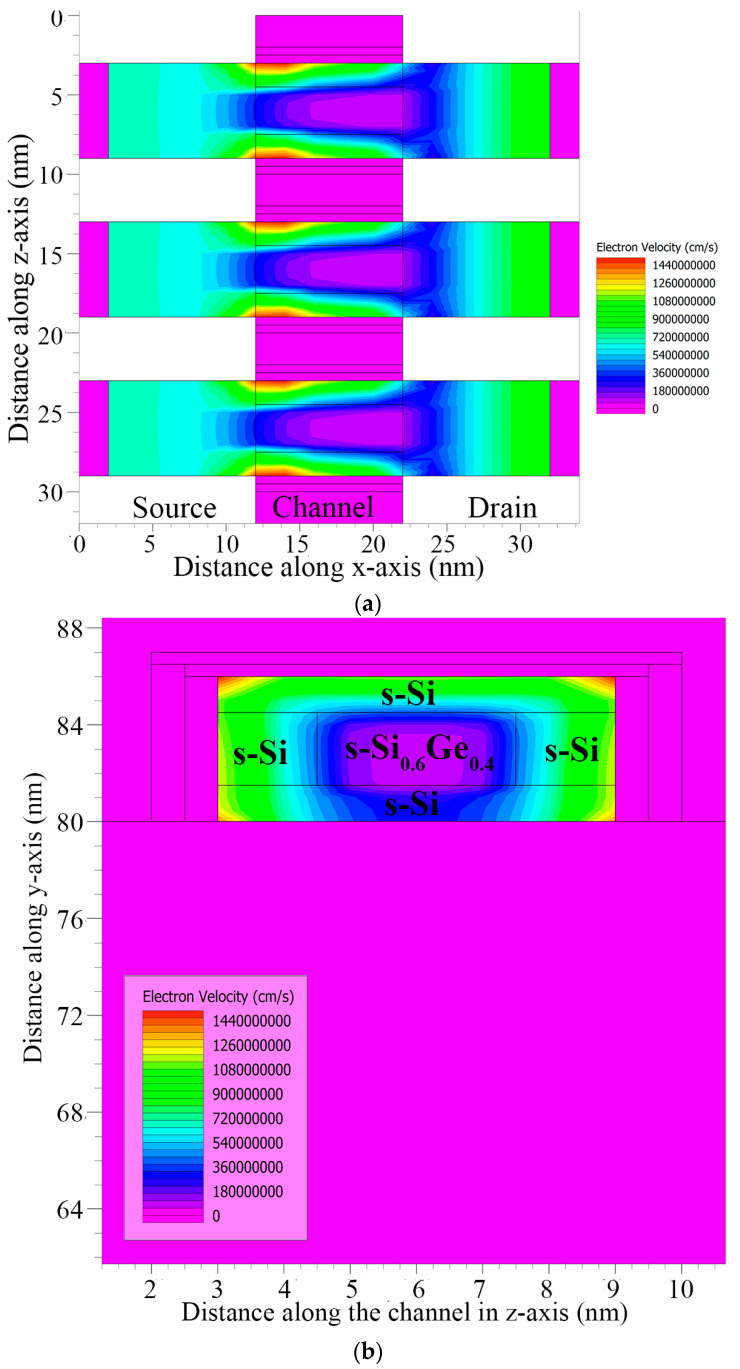
(**a**) Electron velocity contour along lateral direction. (**b**) Electron velocity contour along width.

This improved mobility begins to act on the electron velocity, and the transformation of the TG strained-channel nano-FET device with a gate length of 10 nm is displayed in Figure 13. The electron velocity was analyzed under extreme velocity conditions up to 1000 cm/s, as observed from the contour diagrams visualized in Figure 14a,b for the shorter-gate-length device. This improvement in electron mobility gave increased drive current with extra electron velocity, while maintaining minimal gate-induced drain leakage in the device.

A high electron velocity and electric field in the strained-channel device were detected at the middle s-SiGe layer due to quantum carrier confinement. This is directly attributed to the improved carrier mobility with charge inversion occurring in the narrow- and reduced-channel-length devices with an s-SiGe well in the channel. It should be noted that due to the uniform electric field, the peak electron velocity was observed to be higher in the channel region.

**Figure 15:**
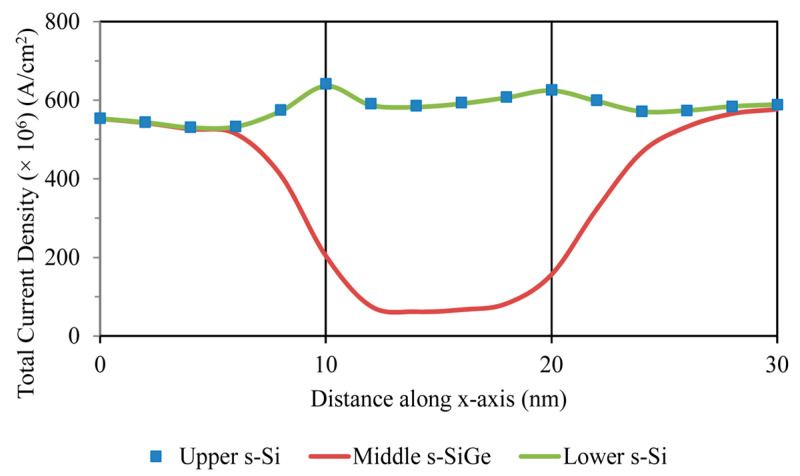
Total current density developed across the channel.

In the strained-channel device, the mobility was improved because of the biaxial strain in the TG layered FinFET, while the SCEs induced an extreme inversion of the total charge density within the channel region; the doping variations at the fin edge and at the gate edge are displayed in Figure 15.

**Figure 16:**
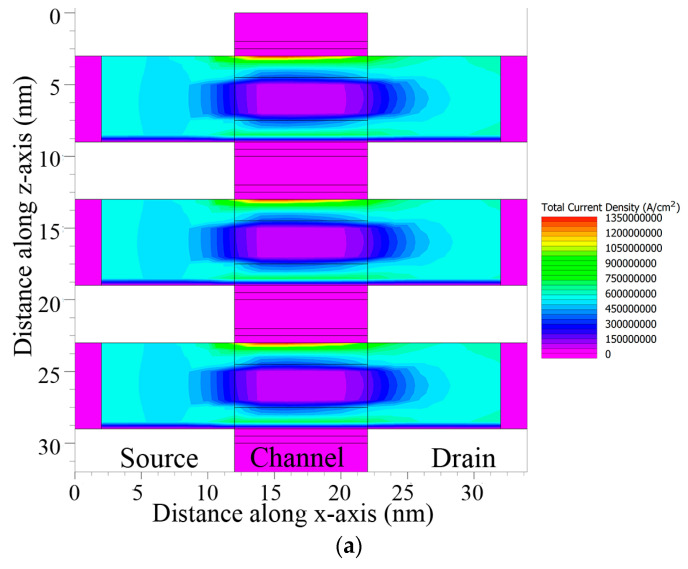

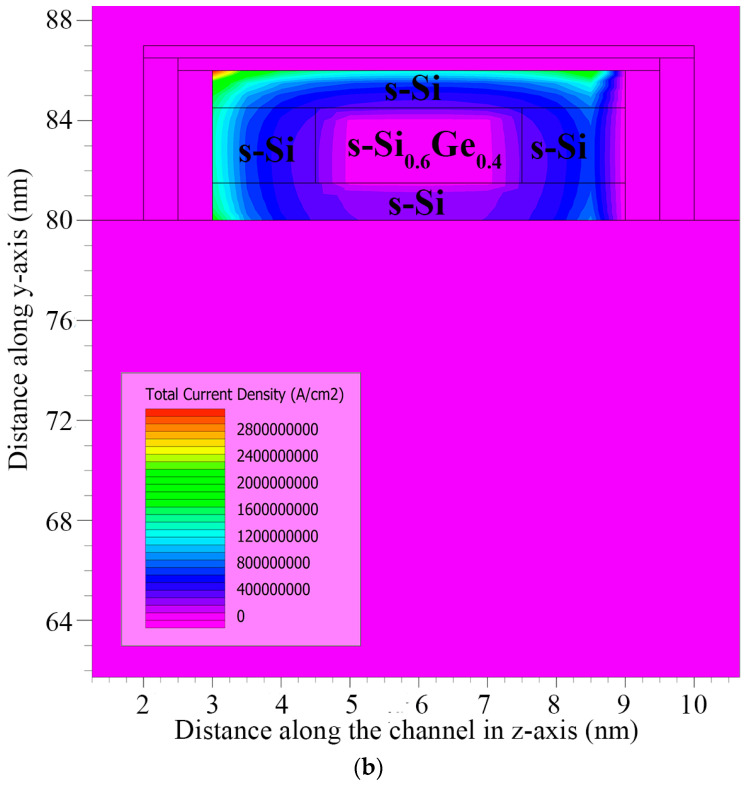
(**a**) Total current density contour along length. (**b**) Total current density contour along width.

With the technically enriched gate control over the device, the current mostly passes through the upper s-Si region of the channel gate, while the middle s-SiGe layer experiences very low current, as depicted in Figure 16a,b. Upon applying external bias via the electric field, the current flows through the strained silicon channel region. From the total current density graph, it can be observed that the current density was higher in the upper s-Si layer than the middle s-SiGe and lower s-Si layers, while the overall total current density was found to be 600 A/cm^2^ from the contours in Figure 16a,b.

## 4. Conclusions

Three-fin HOI n-FinFETs, using three-layered s-Si/SiGe/s-Si as 10 nm length channels, were developed here via the insertion of strain technology to extensively enhance the drive current of the devices. To this end, the gate oxides of the devices were built up in view of high-k stacks like Si_3_N_4_, ZrO_2_, Al_2_O_3_, and HfO_2_, keeping the physical thickness of the high-k material at 0.5 nm. The drain and source were fitted with a height and width of 6 nm. The threshold voltage, drive current, leakage current, *I_on_*/*I_off_* current ratios, subthreshold swing, and DIBL were acquired for all the devices developed here and were compared. It was observed that the TG FinFETs’ performance was improved using strained silicon channels along with high-k stacked dielectrics, instead of a SiO_2_-only gate oxide. These three-fin strained stacked high-k devices provided reduced leakage current and enhanced drive current, particularly when adding HfO_2_-based high-k material. For the SiO_2_-only device, the V_TH_ was 0.197 V, whereas for the stacked SiO_2_ and HfO_2_ device, the V_TH_ was 0.238 V. Similarly, the DIBL for the stacked gate oxide device was 40.99 mV/V, which was quite low in comparison to the DIBL of 59.73 mV/V for the SiO_2_-only device. This proves that the device with HfO_2_ provided optimized results, performed the best, and had improved control over SCEs with very low leakage and improved switching. It was also observed through the cutline views of the band diagram that, due to its electron mobility, electric field, potential, electron velocity, and total current density, the HfO_2_-based stacked device is the most suitable alternative for the future.

## Figures and Tables

**Table 1:** Parameters of the three-fin strained HOI FinFET.

Constraints	Measurements
Drain/source length	10 nm
Channel length	10 nm
Lateral oxide (SiO_2_) thickness	0.5 nm
Physical thickness of the high-k material	0.5 nm
Silicon fin thickness/height	6 nm
Thickness of the strained Silicon in the channel	1.5 nm
Thickness of the SiGe in the channel	3 nm
Substrate + BOX height	80 nm
Channel-doping concentration	10^15^ cm^−3^
Drain/source doping	10^18^ cm^−3^

**Table 2:** Stack (GS)-configured high-k dielectric oxide thickness.

Gate Stack Materials	Physical Thickness of the High-k Material	Dielectric Constant of the High-k Material	EOT (nm)
SiO_2_	-	-	1.0
HfO_2_ + SiO_2_	0.5 nm	25	0.078
ZrO_2_ + SiO_2_	0.5 nm	23	0.085
Si_3_N_4_ + SiO_2_	0.5 nm	7	0.279
Al_2_O_3_ + SiO_2_	0.5 nm	8	0.244

**Table 3:** Comparison of parameters of 10 nm HOI and HOI with high-k stacked 3-fin FinFET.

Parameters	10 nm HOI Three-Fin FinFET	10 nm HOI High-k (HfO_2_) Stacked Three-Fin FinFET
Threshold voltage	0.193 V	0.238 V
On current	466.63 μA/μm	612.24 μA/μm
Off current	1.54 nA/μm	0.94 nA/μm
*I_on_*/*I_off_* current ratios	3.03 × 10^5^	6.51 × 10^5^
Subthreshold swing	71 mV/decade	67 mV/decade
DIBL	61.8 mV/V	40.99 mV/V

## Data Availability

The datasets used and analyzed during the current study are available from the corresponding author on reasonable request.
